# Adult scoliosis treatment combining brace and exercises

**DOI:** 10.1186/1748-7161-8-S2-O8

**Published:** 2013-09-18

**Authors:** Dimitris Papadopoulos

**Affiliations:** 1 Spondylos Laser Spine Lab, Athens, Greece

## Background

Wearing a brace or performing exercises are well-known methods of conservative treatment of adult scoliosis. However, the combination of these two methods has not been investigated.

## Purpose

Based on the practice of wearing a scoliosis brace immediately after exercising to stabilize the stretch effect at the ligaments and the intervertebral disks[1], the purpose of this study was to reveal the importance of wearing the brace after performing exercises. Another goal of this study was to observe any changes in the major problems associated with adult scoliosis: pain, posture and progression of the curve.

## Methods

In April 2009, the study began with 144 adult scoliosis patients: 123 women and 21 men with an average age of 40.8 years (spanning 19– 84 years). The average scoliosis Cobb angle was 40.6° (18°-87.5°). All patients had a degree of pain and had been fitted with a Rigo-Cheneau brace, prescribed for at least eight hours per day after completing minimum 50 minute daily minimum of a combination of Schroth[2] and SEAS methods[3]. Follow-ups were performed every three months for two years. At the end of the 2-year period, the study participants completed a modified Oswestry LBDQ, and satisfaction questionnaire.

## Results

Ten of the 144 patients 26% did not follow the program, 26% followed the program for a few months, 23% inadequately followed the program and 25% accurately followed the program. Following treatment, 68% of the patients had no pain and 67% had improvement in posture and appearance. In 53% of the patients, the Cobb angle improved from 9% to 23%, 18% had no improvement and 29% had an aggravation from 7% to 15%.

## Conclusions and discussion

The combination of brace and exercises yielded excellent results. In general, we noted that, in some cases, only exercises or brace alone decreased pain and/or improved posture, but the most improvement was achieved using both, with the brace worn longer than eight hours per day following the exercises. The combination of Schroth and SEAS methods, selectively to every patient, maximized the results. Since adults have so many obligations, the program was difficult for them to follow, which is why so many abandoned treatment, despite the benefits.
